# Unicompartmental knee arthroplasty, is it superior to high tibial osteotomy in treating unicompartmental osteoarthritis? A meta-analysis and systemic review

**DOI:** 10.1186/s13018-017-0552-9

**Published:** 2017-03-28

**Authors:** Marcel Budhi Santoso, Lidong Wu

**Affiliations:** Department of Orthopedic Surgery, The Second Affiliated Hospital, School of Medicine, Zhejiang University, 88th Jiefang Road, Hangzhou, 310009 Zhejiang Province People’s Republic of China

**Keywords:** High tibial osteotomy (HTO), Unicompartmental knee arthroplasty (UKA), Osteoarthritis, Meta-analysis

## Abstract

**Background:**

Debate remains whether high tibial osteotomy (HTO) or unicompartmental knee arthroplasty (UKA) is more beneficial for the treatment of unicompartmental knee osteoarthritis. The purpose of this study was to compare the functional results, knee scores, activity levels, and complications between the two procedures.

**Methods:**

We performed a systematic review of published literature from August 1982 through January 2017. Fifteen papers reporting three prospective randomized trials were subjected to a meta-analysis.

**Results:**

No significant difference between the two groups was noted with respect to free walking (velocity), knee score, deterioration of the contralateral or patellofemoral knee, or revision rate and total knee arthroplasty. However, UKA produced better outcomes compared to HTO in terms of the functional results, pain assessment, and complications, although patients who underwent HTO tended to have slightly better range of motion.

**Conclusions:**

Valgus HTO provides better physical activity for younger patients whereas UKA is more suitable for older patients due to shorter rehabilitation time and faster functional recovery. Although UKA patients tended to have improved overall long-term outcomes, which may be due to accurate indications and patient selection, both treatment options yielded pleasing results. Therefore, we are unable to conclude that either method is superior.

## Background

The management of degenerative osteoarthritis (OA) aims to provide symptomatic relief and to promote knee function, which may be done conservatively or by means of high tibial osteotomy (HTO) or knee replacement arthroplasty.

HTO is a globally recognized treatment option for medial compartment OA of the knee, particularly for patients who are young and active. This procedure was first conducted in 1958 [1] to correct a varus deformity by lateral mechanical axis relocation [2, 3]. Patients receiving HTO can benefit from natural joint preservation, with physical loading being almost completely unaffected.

Unicompartmental knee arthroplasty (UKA) was first introduced in the 1970s [4] as an alternative to total knee arthroplasty (TKA) or HTO for single-compartment OA. UKA is a joint resurfacing procedure in which the affected degenerative compartment is treated with an implant prosthesis, while the non-affected compartment is preserved. UKA allows knee bone stock preservation and offers patients a less invasive procedure with a faster recovery time [5].

Studies that compare the outcomes of HTO and UKA and their effects are lacking; thus, the relative merits of the two procedures are still under debate. The aim of this study was to evaluate both procedures for the treatment of unicompartmental knee OA using recent reports concerning the indications, functional outcomes, complications, and subsequent revisions to TKA after failed HTO or UKA.

## Methods

### Search strategy

The present study was conducted using the Preferred Reporting Items for Systematic Reviews and Meta-Analyses (PRISMA) statement. A computerized search of electronic databases (MEDLINE, Embase, and Cochrane) for English-language studies, as well as all related published full studies prior to January 2017, was performed using the following keywords to maximize the search sensitivity and specificity: “high tibial osteotomy (HTO),” “unicompartmental knee arthroplasty (UKA),” “unicompartmental knee osteoarthritis,” and “high tibial osteotomy versus unicompartmental knee arthroplasty.”

### Inclusion and exclusion criteria

All retrospective studies and prospective randomized studies that satisfied the search strategy were reviewed and were included in the present analysis if they met the following criteria: studies comparing the outcomes of HTO and UKA that clearly described at least one of the indices investigated in this analysis, articles published in English, and cases with no previous history of knee injury. The title and abstract were examined independently by two reviewers. All disagreements were resolved through discussion until a consensus was reached.

### Data collection

All information regarding participants and clinical outcomes was recorded. Participant data included the number of patients, age, gender, and number of knees treated. The principle outcomes of interest included post-operative functional outcomes, range of motion, velocity, complications, and incidence of revision to TKA. Data were documented independently by two authors after the qualifying studies were selected.

### Quality assessment

The reliability of results depends on the extent to which potential sources of bias have been avoided. To adopt the same method to evaluate all selected studies, two reviewers independently applied the “assessing risk of bias” table to assess the risk of bias in each included study. The following biases were assessed: selection bias, performance bias, attrition bias, detection bias, reporting bias, and other bias. Disagreements were resolved through discussion between the reviewers.

### Statistical analysis

The heterogeneity of this study was determined by documenting the methodological distinctions among several studies by analyzing the data extraction tables. The *I* ^2^ test was used to evaluate statistical heterogeneity; if the *P* value was less than 0.05 and the *I* ^2^ value was less than 50%, a fixed-effects model was selected. However, in cases where these conditions were not satisfied, a random-effects model was adopted [6].

The odds ratio (OR) and associated 95% confidence interval (CI) were used to determine the value of dichotomous data. Continuous data were evaluated by means of the standardized mean difference (STD) and the corresponding 95% CI values using the Mantel–Haenszel method [7].

In all cases, *P* values <0.05 were considered statistically significant. Sensitivity and subgroup analyses were conducted to obtain a solid conclusion and to evaluate the stability of the results. Review Manager (RevMan) version 5.3 for Windows and the Cochrane collaboration were used to interpret the relevant variables and establish the 95% CI.

## Results

### Study characteristic

**Fig. 1:**
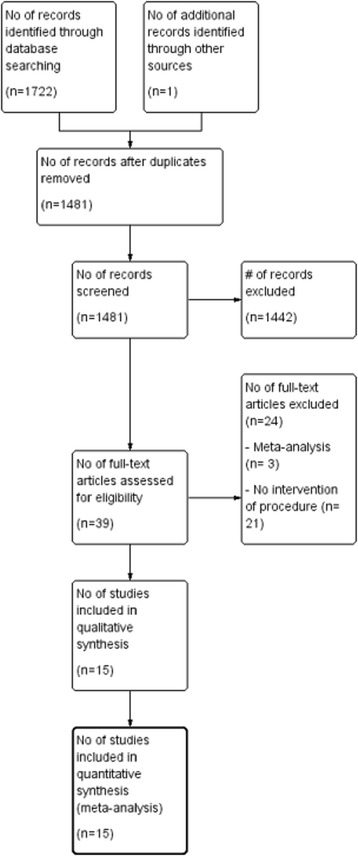
PRISMA Chart

**Fig. 2:**
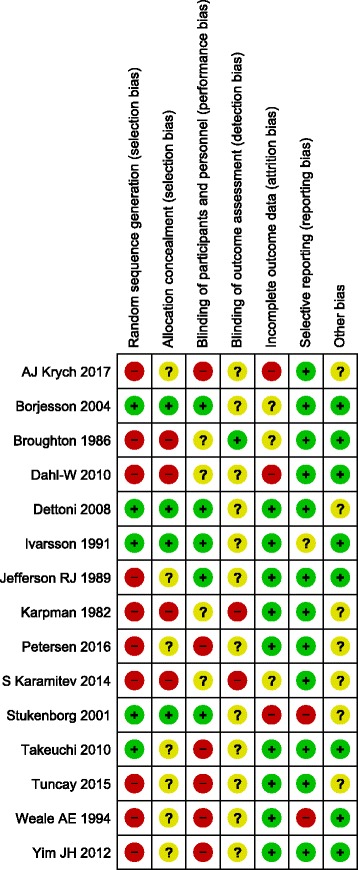
Risk of bias assessment shown in included studies

A total of 1723 titles and abstracts were identified using the search strategies described above, of which 1481 were full-text publications that were then screened based on the inclusion criteria. Thirty-nine studies compared HTO and UKA; however, 24 studies were excluded (Fig. 1). Ultimately, 15 studies [5, 8–21] were selected and included in our analysis, of which only 3 were prospective randomized studies (Fig. 2).

### Population characteristic

Overall, 1013 patients/1041 knees were treated with HTO and 5438 patients/5497 knees were treated with UKA. Patients’ age ranges were 42.7–71 years and 49.2–80 years, respectively. Only 11 studies [5, 8, 9, 12, 13, 15, 17–21] provided patients’ gender: 195 males and 277 females underwent HTO, whereas 182 males and 374 females underwent UKA. The follow-up period ranged from a minimum of 0.5 years to a maximum of 17 years. Eight studies [5, 12, 13, 15, 17, 19–21] reported the indications for inclusion in the study; these were strictly used for isolated medial knee OA with a varus deformity. The inclusion criteria for the other studies were varied or unclear. Six papers [5, 12, 13, 15, 18, 20] reported the cohorts using the Ahlbäck OA score, and three papers [9, 17, 21] used the Kellgreen–Lawrence (K/L) score. All of the included studies described the type of procedure, except one [18] in the HTO group and three [8, 16, 18] in the UKA group. Details are provided in Tables 1 and 2.

**Table 1:** Description of studies included in the meta-analysis

Author	Year	Type of study	Type	Pts	Knee	M/F	Age (years)	HTO type/ UKA model	Follow-up
Karpman et al. [8]	1982	Retrospective	HTO	21	23	18/3	57	CWHTO	2 years
			UKA	19	21	15/4	62	NS	3 years
Broughton et al. [9]	1986	Retrospective	HTO	45	49	11/38	71	CWHTO	7.8 years
			UKA	34	42	11/31	63	St Georg	5.8 years
Jefferson RJ et al.	1989	Prospective	HTO	20	23	NS	57	CWHTO	NS
[10]			UKA	20	24		65	Oxford	
Ivarsson et al. [5]	1991	Prospective	HTO	10	10	4/6	62	CWHTO	1 year
		Randomized	UKA	10	10	4/6	64	Oxford/PCA	0.5 years
Weale et al. [11]	1994	Retrospective	HTO	21	21	NS	74	CWHTO	12–17 years
			UKA	15	15		80	St Georg	12–17 years
Stukenborg et al.	2001	Prospective	HTO	32	32	19/13	67	CWHTO	7.5 years
[12]		Randomized	UKA	28	30	6/22	67	Aesculap	7.5 years
Borjesson et al.[13]	2004	Prospective	HTO	18	18	10/8	63	CWHTO	5 years
		Randomized	UKA	22	22	11/11	63	Brigham	5 years
Dettoni et al. [14]	2008	Prospective	HTO	54		NS	NS	OWHTO	2–4 years
			UKA	56				Accuris	2–4 years
Takeuchi et al. [15]	2010	Retrospective	HTO	24	27	6/18	67	OWHTO	5.1 years
			UKA	18	30	4/14	77	Nakashima	7 years
Dahl-W et al. [16]	2010	Registry	HTO	450		NS	NS	Hemicallotasis	NS
		Review	UKA	4799				Many	
Yim JH et al. [17]	2012	Retrospective	HTO	58	58	7/51	58.3	OWHTO	3.6 years
			UKA	50	50	2/48	60.3	Miller-Galante	3.7 years
S Karamitev et al.	2014	Retrospective	HTO	92	103	47/45	NS	NS	NS
[18]			UKA	65	66	23/42			
Tuncay et al. [19]	2015	Retrospective	HTO	88	93	18/70	52.6	OWHTO + Dome	3 years
			UKA	94	109	15/79	58.7	Oxford	3.5 years
Petersen et al. [20]	2016	Retrospective	HTO	23	23	14/9	58.9	OWHTO	5 years
			UKA	25	25	9/16	60.7	Oxford III	5 years
AJ Krych et al. [21]	2017	Retrospective	HTO UKA	57 183	57 183	41/16 82/101	42.7 49.2	OWHTO + CWHTO Miller–Galante	7.2 years 5.8 years

*year* year of publication, *Type* procedure type, *Pts* patients, *Knee* number of operated knee, *M/F* male/female, *HTO* high tibial osteotomy, *UKA* unicompartmental knee arthroplasty, *CWHTO* close-wedge high tibial osteotomy, *OWHTO* open-wedge high tibial osteotomy, *PCA* porous coated anatomic implant, *NS* not stated

**Table 2:** Summary of data recorded from studies included in meta-analysis

Author	Type	Pts	Knee	E/G results	Pain no/mild	Revision TKA	Complication	Knee	Score	ROM	Velocity	FTA
Karpman et al. [8]	HTO	21	23	11	NS	0	11	NS	NS	NS	NS	NS
	UKA	19	21	19		2	3					
Broughton et al. [9]	HTO	45	49	21	23	10	17	Baily	35.8 ± 7	NS	NS	NS
	UKA	34	42	32	34	3	4		39.6 ± 7.3			
Jefferson RJ et al.	HTO	20	23	NS	NS	5	NS	NS	NS	NS	1.02 ± 0.19	NS
[10]	UKA	20	24			17					0.99 ± 0.21	(+) 3.2°
Ivarsson et al. [5]	HTO	10	10	4	10	NS	NS	Lysholm	78 ± 19	121 ± 11	0.94 ± 0.30	NS
	UKA	10	10	8	10				91 ± 11	112 ± 13	0.93 ± 0.22	
Weale et al. [11]	HTO	21	21	7	9	17	NS	Baily	31	NS	NS	NS
	UKA	15	15	8	12	5			34			
Stukenborg et al.	HTO	32	32	15	NS	10	9	KSS	76 (29–100)	117 (85–135)	NS	(−) 0.25°
[12]	UKA	28	30	13		6	2		74 (31–94)	103 (35–140)		(−) 5.25°
Borjesson et al. [13]	HTO	18	18	18	18	NS	NS	BOA	37 (36–39)	123 ± 0.5	1.13 ± 0.14	NS
	UKA	22	22	22	22				37 (31–39)	123 ± 0.5	1.19 ± 0.15	
Dettoni et al. [14]	HTO	54		50	NS	0	NS	KSS	NS	NS	NS	NS
	UKA	56		53		0			NS			
Takeuchi et al. [15]	HTO	24	27	27	NS	0	2	KSS	89 ± 7.6	146 ± 5.9	NS	170 ± 2.1°
	UKA	18	30	29		2	3		79 ± 6.8	127 ± 16		174 ± 3.8°
Dahl-W et al. [16]	HTO	450		NS	NS	76	NS	NS	NS	NS	NS	NS
	UKA	4799				816						
Yim JH et al. [17]	HTO	58	58	NS	NS	NS	3	Lysholm	89.6 ± 8.7	138.8 ± 4.7	NS	(+) 1.8 ± 1.7°
	UKA	50	50				3		90.3 ± 7.7	130.0 ± 8.8		(−) 1.9 ± 2.2°
S Karamitev et al.	HTO	92	96	83	78	NS	NS	KSS	NS	NS	NS	NS
[18]	UKA	65	66	65	56							
Tuncay et al. [19]	HTO	88	93	NS	NS	0	8	HSS	83.73	NS	NS	NS
	UKA	94	109			3	3		90			
Petersen et al. [20]	HTO	23	23	17	NS	1	2	HSS	NS	NS	NS	NS
	UKA	25	25	21		1	1					
AJ Krych et al. [21]	HTO	57	57	NS	NS	13	NS	Lysholm	80.2 ± 11.8	NS	NS	(+) 1.3 ± 2.4°
	UKA	183	183			11			90.0 ± 11.0			NS

*Type* procedure type, *Pts* patients, *Knee* number of operated knee, *E/G* excellent, good result, *Pain* pain assessment, *ROM* range of motion, *Velocity* free walking speed, *FTA* femoro-tibial angle, *Baily* Baily knee score, *Lysholm* Lysholm knee score, *KSS* Knee Society score, *BOA* British Orthopaedic Association score, *HSS* Hospital for Special Surgery score, *(+)* valgus, *(−)* varus, *NS* not stated

### Meta-analysis

**Fig. 3:**
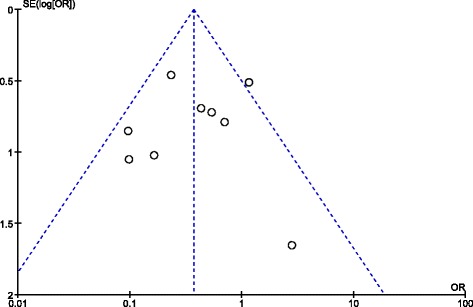
Funnel plot to assess small study exclusion/publication bias

Because the measurement time points varied among studies, nearly all results reported here reflect the pooled data without period stratification. In addition, not all studies presented the essential data, introducing a potential bias to this study. Upon analyzing statistical heterogeneity, seven outcomes showed substantial heterogeneity (*I* ^2^ values >50%) and were therefore interpreted with caution (Table 3). There was limited evidence of a publication bias, with a broad symmetrical funnel plot assessing the primary outcomes (excellent/good results) (Fig. 3).

**Table 3:** Result of the meta-analysis

Outcome	Studies	Sample size		Effect estimate	P	Effect estimate	Heterogeneity	
		HTO	UKA	Odds ratio (95% CI)		STD (95% CI)	*I* ^2^ (%)	Chi^2^ (*P*)
Pain assesment (no/mild)	5	194	155	0.34 [0.13, 0.91]	0.03		61	0.08
Excellent/good (E/G) result	10	353	317	0.37 [0.24, 0.58]	<0.00001		39	0.11
Excellent/good result (medial OA/varus)	5	110	117	0.75 [0.37, 1.52]	0.43		19	0.29
Subgroup: E/G CWHTO-UKA	6	153	140	0.36 [0.21, 0.61]	0.01		56	0.06
Subgroup: E/G OWHTO-UKA	3	104	111	0.70 [0.26, 1.91]	0.49		0	0.66
Knee score	7	262	317		0.11	−0.21 [−0.47, 0.05]	51	0.05
Lysholm knee score	3	92	126		0.08	−0.53 [−1.12, 0.06]	71	0.03
Knee Society Score (KSS)	2	59	60		0.59	0.10 [−0.26, 0.46]	0	0.88
Deterioration of contralateral	2	107	92	2.24 [0.30, 16.72]	0.43		74	0.05
Deterioration of patellofemoral	2	107	92	2.01 [0.67, 6.04]	0.21		0	0.57
ROM	5	145	142		0.008	0.78 [0.21, 1.36]	80	0.0005
Velocity	3	51	51		0.66	−0.09 [−0.48, 0.30]	0	0.44
Complication	7	305	307	3.08 [1.76, 5.39]	<0.0001		7	0.37
Revision rate	11	880	5361	1.18 [0.54, 2.58]	0.68		74	<0.0001

*HTO* high tibial osteotomy, *UKA* unicompartmental knee arthroplasty, *P* p value, *E/G* excellent, good result, *OA* osteoarthritis, *Varus* varus deformity, *STD* Std mean difference, *CI* confidence interval

### Primary outcome

**Fig. 4:**
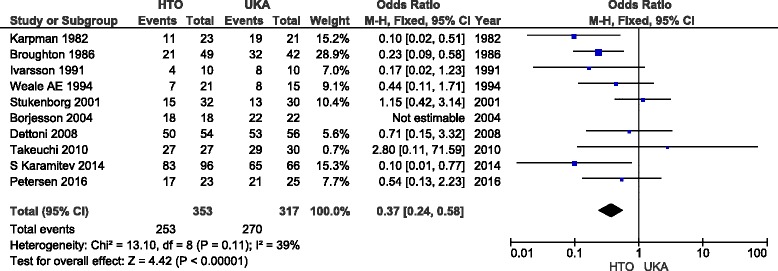
Forrest plot of ten studies presenting data about primary outcome (excellent/good) result

The analysis of 10 studies [5, 8, 9, 11–15, 18, 20] yielded a statistically significant difference between HTO and UKA regarding excellent/good results (*p* < 0.001; OR = 0.37; 95% CI = 0.24, 0.58; Fig. 4). Among them, five studies [5, 12, 13, 15, 20] provided clear support for medial OA, specifically in cases of varus deformity; however, the difference was not significant (*p* = 0.43; OR = 0.75; 95% CI = 0.37, 1.52). Moreover, the subgroup analysis for the HTO group included opening [14, 15, 20] and closing-wedge [5, 8, 9, 11–13] procedures; these yielded differing results, with *p* values of 0.49 and 0.01, respectively, compared to the UKA group.

### Pain assessment

Five studies [5, 9, 11, 13, 18] reported post-operative results for pain assessment. Patients in the UKA group tended to have better results. According to our analysis, the difference was significant (*p* = 0.03; OR = 0.34; 95% CI = 0.13, 0.91).

### Deterioration

Based on the available data, only two studies [9, 17] included information on deterioration. However, the difference for contralateral deterioration was not significant (*p* = 0.43; OR = 2.24; 95% CI = 0.30, 16.72), nor was the difference for patellofemoral deterioration (*p* = 0.21; OR = 2.01; 95% CI = 0.67, 6.04).

### Range of motion (ROM)

Our analysis revealed better flexion and extended ROM in the HTO group compared to the UKA group in five studies [5, 12, 13, 15, 17], with *p* values <0.01 (STD = 0.78; 95% CI = 0.21, 1.36).

### Free walking speed (velocity)

Only three studies [5, 10, 13] compared the free walking speed between HTO and UKA patients; these showed no significant difference (*p* = 0.66; STD = −0.09; 95% CI = −0.48, 0.30).

### Knee score

Seven studies [5, 12, 13, 15, 17, 19, 21] used various scoring systems to compare knee scores between the two procedures. Although no statistically significant difference was found (*p* = 0.11; STD = −0.21; 95% CI = −0.47, 0.05), the UKA group exhibited better functional results. Our study also analyzed the Lysholm knee score [5, 17, 21] and Knee society score (KSS) [12, 15], which showed no significant differences (*p* = 0.08 and 0.59, respectively).

### Complication

Generally, more complication were noted after a valgus HTO with significant difference found between the two groups (*p* < 0.001; OR = 3.08; 95% CI = 1.76, 5.39), reflecting results from seven studies [8, 9, 12, 15, 17, 19, 20] with 559 patients.

### Revision

**Fig. 5:**
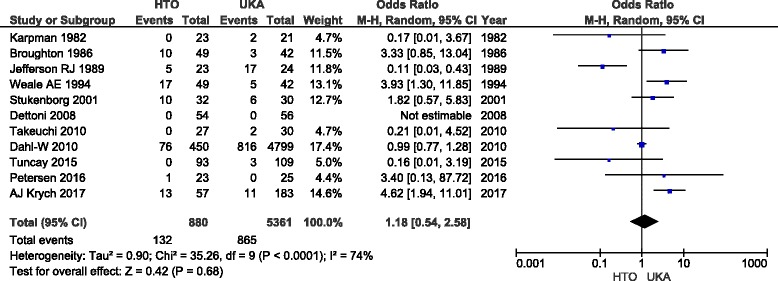
Forrest plot of ten studies presenting data about revision rate

Eleven studies [8–12, 14–16, 19–21] with 6241 patients reported revisions. The pooled data showed no significant difference between HTO and UKA in terms of revision rate (*p* = 0.68; OR = 1.18; 95% CI = 0.54, 2.58; Fig. 5).

## Discussion

OA affects any or all three compartments of the knee. However, one third of patients are afflicted in only one of these compartments, many of them having a medial compartment disorder [22].

The purpose of surgery for unicompartment OA is to reduce pain, restore function, and improve the patient’s quality of life. The most important finding of this study was that both HTO and UKA are satisfactory operative treatment options for symptomatic medial knee OA.

Patient selection is generally stricter for individuals undergoing HTO than for those receiving UKA. However, medial knee arthritis patients selected for HTO experience many benefits. Ideal indications for HTO include (1) young and active patients (age <65 years) [23, 24], (2) normal-range body mass index (BMI) [25], (3) mild articular destruction (no more than grade 2 Ahlbäck classification), (4) no patellofemoral arthrosis [26], and (5) good ROM and a stable joint [27].

Age, BMI, and pre-operative state OA are key factors that optimize clinical outcomes and survival in patients undergoing HTO. Previous studies have reported that a pre-operative BMI higher than 27.5 is a significant risk factor for early failure [25], and patients with BMI over 30 exhibit significantly lower KSS and WOMAC scores 5 years after HTO [28]. Moreover, HTO is not advisable for patients older than 65 years due to the 7.6% increased risk per year of age and the 1.5-fold relative risk of failure compared to younger patients [29].

HTO and UKA share similar indications that include the following: age 55–65 years, moderately active, non-obese, presenting with mild varus malalignment and moderate unicompartmental arthrosis, no joint instability, and good ROM [30].

Indications for UKA are broadening after reports of promising mid- and long-term results, which include isolated medial or lateral compartment OA, osteonecrosis of the knee, age over 60 years, weight under 82 kg, and an ideal ROM of 90 with fewer than 5° flexion contractures. Contraindications include high activity, age under 60 years, and inflammatory arthritis [11, 30, 31].

Our analysis demonstrated a significant difference in outcomes between UKA and HTO patients, with the former showing better functional results (excellent/good results), and the latter better ROM. This discrepancy was correlated with knee score and ROM, indicating the possibility of additional impacts on the functional results.

Earlier publications reported a valgus deformity treated with either procedure. In our opinion, the clinical results for patients with a surgically treated valgus deformity, by either arthroplasty or osteotomy, cannot be compared to results for patients with a varus deformity. Major differences between medial and lateral UKA, as well as between varus and valgus osteotomy have been noted [4, 9, 32–34]. Therefore, our analysis showing excellent/good functional results focused only on studies with a strict inclusion criterion of medial knee OA with a varus deformity; analysis of such cases showed no significant difference between the two procedures. The subgroup analysis yielded similar findings and revealed favorable results in the UKA group relative to closed-wedge HTO (CWHTO) patients; however, these results were not noted when comparing open-wedge HTO (OWHTO) and UKA. CWHTO was the main treatment method for HTO in the past, but OWHTO was recently reported to yield good or excellent results, owing to improvements in surgical techniques and implant stability [35, 36]. However, recent meta-analyses comparing CWHTO and OWHTO did not report superiority of OWHTO over CWHTO [37, 38].

A greater change in ROM was noted in the UKA group relative to the HTO group due to a lower pre-operative score [5]. Takeuchi et al. [15] reported that OWHTO is a more appropriate treatment method for active patients who require good ROM of the knee. The unsatisfactory results of the HTO group were mostly due to an insufficient deformity correction. Previous studies reported that optimal results can be achieved if the mechanical alignment is adjusted to 7° [39]. Nevertheless, the ultimate post-operative valgus position is technically challenging to achieve.

Free walking speed (velocity) has been proven both a reliable and a valid indicator to evaluate treatment outcomes in knee OA patients [40, 41]. Our meta-analysis found no significant difference between the two procedures in terms of velocity (*p* = 0.66), although Fu et al. [42] reported otherwise (*p* = 0.05). However, given that both studies used the same literature to arrive at this outcome, differing results were not expected. It is also important to note that Jefferson et al. [10] assessed the velocity outcome of three operative methods (HTO, UKA, and TKA), with post-operative results reported as 1.02 ± 0.19, 0.99 ± 0.21, and 0.81 ± 0.19 m/s, respectively. However, Fu et al. [42] included the TKA results (0.81 ± 0.19) in their analysis of the HTO group, which may suggest an inaccuracy. Therefore, our results are more accurate and reliable.

Our analysis revealed that free walking speed was improved after both HTO and UKA but with an equivalent rise in the UKA group. Borjesson et al. [13] stated that, compared to HTO patients, UKA patients had a greater increase in free walking speed, with results 5 years after surgery that were highly similar to the walking speed of healthy people of the same age group [43]. Moreover, both procedures resulted in an almost normal gait pattern.

Ivarsson et al. [5] showed that UKA patients have better muscle strength than do HTO patients 6 months post-operatively, but the 12-month post-operative results were similar. One explanation for this finding is that rehabilitation of UKA patients normally begins earlier, whereas HTO patients usually undergo an immobilization period. Moreover, HTO patients may require a longer time to adapt due to greater changes in post-surgical leg alignment.

Regarding the progression of knee OA, our analysis showed that the OR of the risk of contralateral and patellofemoral deterioration did not differ between groups, although the HTO group tended to exhibit this problem. One logical explanation is that this phenomenon is due to the overcorrection to unleash the medial compartment during the procedure, thus suppressing the lateral compartment and leading to deterioration. Overcorrection of more than 6° was associated with progressive degeneration of the lateral compartment [44]. In addition, OWHTO above the tibial tubercle can have adverse effects on patellofemoral articulation [2, 45, 46]. Yim et al. [17] compared OWHTO and UKA patients and reported that two cases of UKA showed patellofemoral joint OA compared to three cases of OWHTO.

Compared to UKA, the chance of post-operative complications is greater after an osteotomy [39]. Our analysis revealed a significant difference in such complications between HTO and UKA patients, supporting previous studies and a meta-analysis by Spahn et al. [47]. Among all included studies in this present study, five trials applied OWHTO, seven trials used CWHTO, and one study used hemicallotasis. OWHTO is considered safe and easy [21, 48, 49] based on the assumption that CWHTO may be associated with a higher incidence of complications, especially peroneal nerve paralysis. Despite improved surgical techniques and implant design, previous studies have reported complications after UKA, such as loosening of the tibial or femoral component or osteoarthritic changes in the development of the lateral compartment due to antero–posterior instability of the knee, which leads to rapid wearing of the polyethylene insert [11, 21]. In the HTO group, most complications were associated with an intra-articular fracture, nonunion, infection, and peroneal nerve palsy.

TKA is defined as a clear end-point after both HTO and UKA. Medial UKA patients tend to require revision sooner [21], with a mean of 8.2 years compared to a mean of 9.7 years for valgus HTO patients [47]. Barrett and Scott [50] reported 29 unsuccessful UKA revisions to TKA and observed that the mechanism of failure was loosening in 55% of cases and degeneration advancement of the remaining compartments in 31% of patients. Technical errors during the primary UKA and poor selection of patients contributed to 66% of failures.

Cross et al. [51] examined the operative time and found that revision to TKA in HTO patients required more time compared to that for UKA patients, which could be because the HTO procedure is complicated by difficulties in obtaining an acceptable exposure, removing retained hardware, achieving correct tibial component positioning, scarring, and additional challenges with ligamentous balancing that have been reported to result from a prior HTO. The major technical difficulty in the revision UKA group was handling the bony defects on both the tibial and femoral sides. Significantly thicker polyethylene inlays were required during the revision of UKA to TKA compared to primary TKA [52], and the UKA group required substantially more osseous reconstruction (77%) compared to the HTO group (20%) [30].

Consistent with the previous meta-analysis [42], the present study also failed to identify any significant difference in the revision rate between the two procedures. Although both groups exhibited higher revision rates over time with deteriorated clinical outcomes, the risk of revision of primary UKA declined with age. The 10-year revision rate was nearly 24% in patients aged less than 55 years, threefold higher than that in those aged 55 years and older [16].

Robertsson et al. [53] reported that hospitals that perform 23 or more UKAs per year have a 1.6-fold lower revision rate compared to those who perform fewer than 23. Therefore, routine patient selection and good surgical skills are believed to influence the results of the UKA procedure; this principle may also apply to HTO.

Several limitations of this study should be noted. First, a controlled randomized trial is challenging due to ethical concerns. The present meta-analysis included only three randomized controlled trials of the 15 studies and the patients enrolled for HTO tended to be younger than those enrolled for UKA. Although most studies reported good numbers, the use of diverse analyzing systems and methods can lead to difficulties comparing and assembling the outcomes, as well as inability to evaluate essential items such as radiographic changes due to inadequate data. Moreover, the current analysis showed that UKA and HTO are distinct in terms of their techniques and indications for patients with medial unicompartmental OA. Finally, the small patient population made it difficult to compare the two procedures and arrive at a conclusion regarding the clinical outcomes.

## Conclusions

In conclusion, valgus HTO is a technically challenging procedure but provides younger OA patients with good physical activity. On the other hand, UKA is more suitable for older patients, as it provides a greater quality of life with a shorter rehabilitation time required before full weight bearing, fewer perioperative complications, and faster functional recovery compared to HTO.

Accurate identification of indications, including age, level of activity, grade of OA and ROM of the knee, and careful patient selection are essential for all OA patients. Nevertheless, with advancements in surgical techniques, implant design and patient selection, UKA has become a more reliable and effective procedure.

Finally, although UKA patients tended to have improved overall long-term outcomes, both treatment options offered pleasing results, and no significant evidence supports one method over the other. Additional well-designed and large-scale clinical trials and systematic reviews are necessary to confirm the findings presented here.

## Abbreviations

BMI: Body mass index
CI: Confidence interval
CWHTO: Closing-wedge high tibial osteotomy
HTO: High tibial osteotomy
KSS: Knee society score
OA: Osteoarthritis
OR: Odds ratio
OWHTO: Opening-wedge high tibial osteotomy
ROM: Range of motion
STD: Standardized mean difference
TKA: Total knee arthroplasty
UKA: Unicompartmental knee arthroplasty
